# Bacteria isolated from biodiverse Mediterranean island habitats yield a large array of biopesticidal metabolites against mosquito larvae

**DOI:** 10.1128/aem.00966-25

**Published:** 2025-07-07

**Authors:** Martyn J. Wood, Katerina Kanelli, Juan J. Silva, Joel C. Couceiro, Andronikos Papadopoulos, F. Elektra Maridaki, Maria Mazavli, Inga Siden-Kiamos, John Vontas, George Dimopoulos

**Affiliations:** 1 Institute of Molecular Biology and Biotechnology, Foundation for Research and Technology-Hellas (IMBB-FORTH) 69153, Heraklion, Crete, Greece; 2 Department of Biology, University of Crete 37777, Rethimno, Greece; 3 Pesticide Science Laboratory, Department of Crop Science, Agricultural University of Athens 68995, Athens, Greece; 4 W. Harry Feinstone Department of Molecular Microbiology and Immunology, Bloomberg School of Public Health, Johns Hopkins University 25802, Baltimore, Maryland, USA; UMR Processus Infectieux en Milieu Insulaire Tropical, Ste. Clotilde, France

**Keywords:** bacteria, biopesticide, insecticide, mosquito, larvicide

## Abstract

Microbes are prolific in synthesizing diverse compounds in response to varying environmental conditions, many of which have applications in insect pest control. Fewer than 20,000 of the estimated millions of bacteria have been named, and only a small subset of known bacteria has led to the development of some of today’s most critical pesticides, so the potential for discovering new insecticidal compounds from bacteria remains vast. We collected 1,663 bacterial isolates from environmental samples across diverse habitats in Crete. Screening 788 of these for larvicidal activity against *Culex pipiens molestus* identified 108 unique larvicidal isolates, with 37 causing 100% mortality in less than 3 days; these top hits represent 20 genera, many of which are novel in the arena of candidate biopesticides. Our 37 top isolates mediated their larvicidal activity after inactivation, indicating that metabolites or proteins rather than infections mediated mortality. Three new isolates of *Chryseobacterium* and *Pseudomonas* produce insecticidal factors that achieved 100% mortality within 48 h. While non-polar extractions were primarily responsible for larvicidal activity, promising polar extractions also showed potential as novel biopesticidal agents.

**IMPORTANCE:**

The development of environmentally friendly, biological solutions to combat insecticide resistance and contribute to effective vector management strategies has become increasingly important in recent decades. The study presented herein describes the discovery of a number of novel insecticidal-metabolite-producing bacteria demonstrating high efficiency and rapid action. Furthermore, some basic chemistry of the active metabolites is investigated, facilitating accelerated development of the newly discovered bacterial isolates and metabolites. In particular, three isolates: a *Chryseobacterium* and two *Pseudomonas* species, each of which are highly potent larvicidal agents, and whose active metabolites are of differentiated base chemistry. The impact of this work is to heighten awareness of the vast potential of bacteria as a source of novel tools for insect control strategies, and it is hoped that the work will lay the foundation for the development of several new tools that demonstrate high efficacy as applied biopesticides.

## INTRODUCTION

Mosquito-borne diseases continue to account for over 700,000 deaths annually, and mosquito control remains the most important control method. However, growing insecticide resistance among key vector genera such as *Aedes* (1), *Anopheles* (2, 3), and *Culex* (4, 5), even within Europe (6), together with environmental and health concerns (7, 8), have led to regulatory restrictions and the removal of more than 80% of all synthetic insecticides from the market in the past two decades (9). Political and environmental pressures, particularly in the EU, now encourage biological and sustainable alternatives for pest control as part of the EU Green Deal.

Fungal microbial biological control agents (BCAs) have shown promise in recent decades (10–12) and are widely used in agriculture (13, 14). However, entomopathogenic bacteria are less utilized outside of mosquito control, while the most effective against mosquitoes are *Bacillus thuringiensis* (specifically *B.t*. var. *israelensis* and *B.t*. var. *kurstaki*) and *Bacillus sphaericus* (15). However, field application of microbial biopesticides remains challenging due to inconsistent efficacy (16), high cost, and the need for frequent reapplication (17, 18). These challenges necessitate ongoing discovery and development of novel and diverse biocontrol agents.

While the search for novel insecticidal compounds from microbes has a long history, it has still only yielded a limited number of control agents, among which are Spinosad (19) and various Bt toxins (20, 21). The discovery of microbial biopesticides has often focused on well-studied isolates and regions while screening in Europe, particularly the Mediterranean region, has been limited.

Here, we have conducted an extensive survey and screening of microbial isolates on the island of Crete in Greece, identifying several promising bacteria that exert insecticidal activity against the larvae of *Culex pipiens molestus* through their produced metabolites. Many of these isolates show strong potential for future development as BCAs, with three select isolates producing metabolites capable of causing 100% mortality within 24 h exposure.

## RESULTS

### Bacterial discovery and library generation

**Fig 1:**
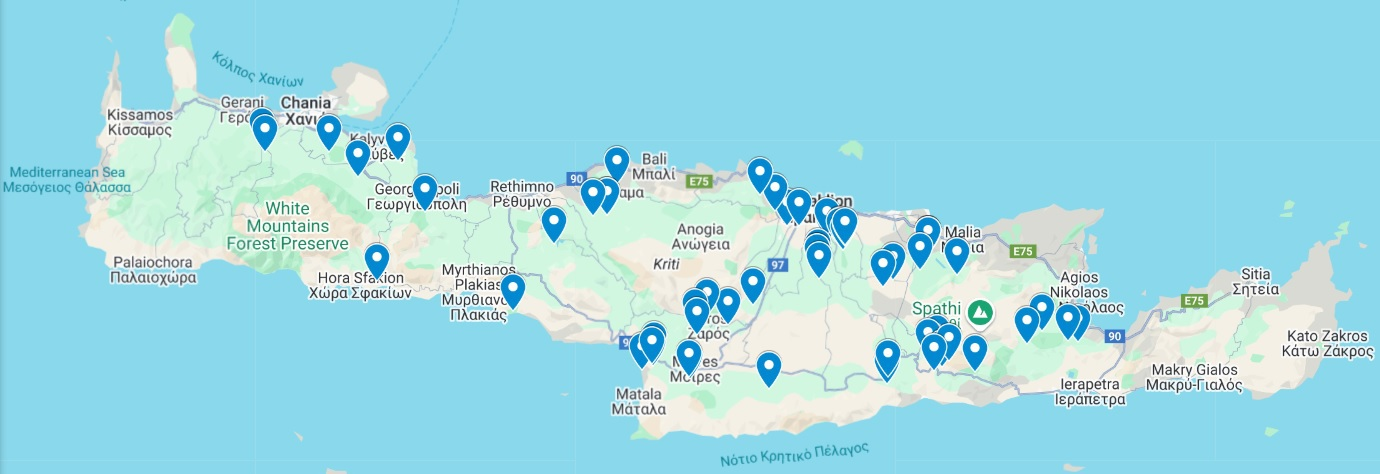
Map highlighting the 65 locations across the island of Crete, Greece, where a total of 195 soil, root rhizosphere, bacterial endophyte, insect, water, and plant matter samples were collected. These 65 locations resulted in the generation of 1,663 morphologically distinct bacterial samples. Map sourced from Google Maps (n.d.): Map of Crete with locations of microbial collection sites plotted as destinations. Retrieved from: https://www.google.com/maps/d/viewer?mid=1qwUBrHat8De79XiH5FmbjNxM3zM6mI0&ll=35.29660049600868%2C24.86015029635919&z=9 on 20 May 2025 using Google Map Data© 2025.

We collected 186 environmental samples from 65 diverse locations across the island of Crete (Fig. 1). Our sampling strategy largely focused on terrestrial environments likely to harbor bacteria with insecticidal activity (22, 23). Subterranean and aquatic habitats were prioritized for their relative stability and sheltering from environmental fluctuations, which support a greater microbial diversity than surface habitats (24). Rhizosphere soils, plant tissues (to isolate endophytes), and water sources were also targeted since plants are known to recruit insecticidal microbes in response to pest damage (22, 25). We also collected dead insects to identify potential microbes directly relevant to pest control; however, most of these specimens were too degraded for morphological identification beyond the family level, with the exceptions of one mosquito larva, one aphid, and one true fly.

**Fig 2:**
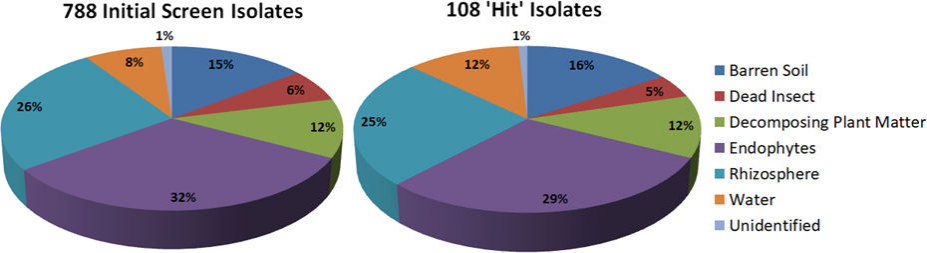
Graphical visualization of the % makeup for sources of each of the 788 isolates included in the initial high-throughput screen and of the 108 bacteria that were found to display insecticidal attributes against *Culex pipiens molestus* larvae.

Our 186 samples can be broadly categorized into: top soils (*n* = 31), rhizosphere soils (*n* = 41), internal plant endophytes (*n* = 54), water samples (*n* = 20), dead insects (*n* = 13), and other sources (*n* = 27) (Fig. 2). Culturing these samples via PBS immersions on LB and Czapek media produced 1,663 morphologically distinct bacterial colonies, which represented our comprehensive microbial library.

### Coarse screening of 788 live bacterial isolates against *C. pipiens molestus* larvae

To investigate specific properties relating to the mode of action and nature of the bacteria-produced insecticidal factors, variations of a mortality bioassay protocol were implemented to distinguish bacteria that: (i) kill through sepsis or infection, (ii) kill through metabolite production, (iii) produce insecticidal metabolites that are secreted into the culture medium, and (iv) produce either polar, non-polar, or a complex mixture of insecticidal metabolites. We selected 788 bacterial isolates based on colony morphology and pigmentation on LB agar plates, because unusual growth rates and pigmentation are often associated with metabolite production that can exhibit diverse biological activities. In the high-throughput screening, third- or fourth-instar *Cx. pipiens* larvae were exposed to a 1 mL water solution containing a 10×-diluted bacterial culture, and mortality was monitored over 7 days and this yielded 108 isolates that caused 100% larval mortality (Fig. S1, Panels: A–L). The 37 isolates that achieved complete larval mortality within 3 days were selected as primary “hits” for further screening (Table 1).

**TABLE 1:** List of the 37 top bacterial isolates, identified by the high-throughput live bacterial screen, as having a strong indication of insecticidal properties with fast action^*a*^

Isolate code	LT_100_ (days)	Mean larval survival time (days)	Isolate code	LT_100_ (days)	Mean larval survival time (days)	Isolate code	LT_100_ (days)	Mean larval survival time (days)
OTN78-C5	1	1	OTN134-CD2	3	2.5	OTN131-CD2	7	2.833
OTN84-CD3	3	1.334	OTN143-C3	3	2.5	OTN144-C3	5	2.833
OTN166-C1	3	1.667	OTN146-C4	3	2.5	OTN147-C4	7	2.833
OTN167-C2	4	1.667	OTN189-C6	5	2.5	OTN3-C1	7	3
OTN167-C9	4	1.667	OTN15-C6	3	2.667	OTN11-CZ3	6	3
OTN1-C11	2	1.667	OTN85-CD2	4	2.667	OTN30-CD6	5	3
OTN85-C3	3	2	OTN88-C3	5	2.667	OTN30-CD7	5	3
OTN141-CD4	4	2	OTN111-C7	4	2.833	OTN49-CD2	3	3
OTN147-C3	2	2	OTN63-C5	4	2.833	OTN82-C2	4	3
OTN184-C8	3	2	OTN72-C2	4	2.833	OTN82-C8	6	3
OTN144-CD4	3	2.167	OTN78-CD1	5	2.833	OTN85-C8	5	3
OTN141-C1	5	2.333	OTN110-CD3	5	2.833	OTN115-C6	5	3
OTN77-C6	5	2.5						

^*a*^ All results are derived from live bacterial cultures inoculated into water containing third- and fourth-instar *Culex pipiens molestus* larvae. The table gives the isolate codes for each of the bacteria according to the IMBB FORTH microbial library, alongside the lethal time 100 (LT_100_) values for each and the mean survival time for all insects included in each treatment.

Initial statistical analysis revealed no significant correlations between the original habitat type of the bacterial isolates and their insecticidal properties. The proportion of insecticidal bacteria (*N* = 108) isolated from each habitat type was consistent with the overall proportion of bacteria isolated from that habitat (*N* = 788) (Fig. 2).

### Identification of bacteria via ribosomal protein gene 16S sequencing

Identification of the bacterial genera was performed for the 37 selected larvicidal isolates (Table 2), indicating that they belonged to 20 different genera. The most abundant were *Pseudomonas* (eight isolates), *Stenotrophomonas* (four), *Microbacterium* (three), and *Micrococcu*s (three). Three genera were represented by two isolates each: *Curtobacterium*, *Bacillus*, and *Pantoea*. There were 13 genera represented by a single isolate (*Herbiconiux*, *Mycobacterium*, *Pseudoxanthomonas*, *Variovorax*, *Chryseobacterium*, *Kocuria*, *Exiguobacterium*, *Planococcus*, *Rhodococcus*, *Dermacoccus*, *Paracoccus*, and *Streptomyces*) (Table 2).

**TABLE 2:** Genus identification of the top 37 bacterial hits based on Sanger sequencing of the 16S region of their rRNA^*a*^

Isolate code	Genus code	Region amplified	Length (bp)	Genus	KIR	mKIR
OTN1-C11	Cr1	V1–V9	1,173	*Chryseobacterium*	Y	N
OTN3-C1	St1	V1–V9	1,453	*Stenotrophomonas*	N	N
OTN11-CZ3	Ps1	V1–V9	1,436	*Pseudomonas*	Y	Y
OTN15-C6	St2	V1–V9	1,448	*Stenotrophomonas*	N	N
OTN30-CD6	Mc1	V1–V9	1,423	*Micrococcus*	N	N
OTN30-CD7	Pc1	V1–V9	1,335	*Paracoccus*	N	N
OTN49-CD2	Px1	V1–V9	1,456	*Pseudoxanthomonas*	N	N
OTN63-C5	He1	V1–V9	1,433	*Herbiconiux*	N	N
OTN72-C2	Ps2	V1–V9	1,436	*Pseudomonas*	Y	Y
OTN77-C6	Ko1	V1–V9	1,422	*Kocuria*	N	N
OTN78-C5	Ps3	V1–V9	1,448	*Pseudomonas*	Y	Y
OTN78-CD1	Ps4	V1–V9	1,407	*Pseudomonas*	Y	Y
OTN82-C2	Ba1	V1–V9	1,464	*Bacillus*	Y	Y
OTN82-C8	Sm1	V1–V9	1,426	*Streptomyces*	Y	Y
OTN84-CD3	St4	V1–V9	1,449	*Stenotrophomonas*	N	N
OTN85-C3	Cu1	V1–V9	1,421	*Curtobacterium*	N	N
OTN85-C8	Mb1	V1–V9	1,419	*Microbacterium*	Y	N
OTN85-CD2	Pl1	V1–V9	1,171	*Planococcus*	N	N
OTN88-C3	Rh1	V1–V9	1,422	*Rhodococcus*	Y	Y
OTN110-CD3	My1	V1–V9	1,390	*Mycobacterium*	N	N
OTN111-C7	Ps5	V1–V9	1,448	*Pseudomonas*	Y	Y
OTN115-C6	Ps6	V1–V9	1,447	*Pseudomonas*	Y	Y
OTN131-CD2	Pb1	V1–V9	1,429	*Paenarthrobacter*	N	N
OTN134-CD2	Pa1	V1–V9	1,454	*Pantoea*	Y	N
OTN141-C1	Pa2	V1–V9	1,455	*Pantoea*	Y	N
OTN141-CD4	St4	V1–V9	1,203	*Stenotrophomonas*	N	N
OTN143-C3	Ex1	V1–V9	1,146	*Exiguobacterium^b^*	N	N
OTN144-C3	De1	V1–V9	1,395	*Dermacoccus*	N	N
OTN144-CD4	Mb2	V1–V9	1,389	*Microbacterium*	Y	N
OTN146-C4	Mc2	V1–V9	1,415	*Micrococcus*	N	N
OTN147-C3	Mc3	V1–V9	1,417	*Micrococcus*	N	N
OTN147-C4	Cu2	V1–V9	1,420	*Curtobacterium*	N	N
OTN166-C1	Mb3	V1–V9	1,390	*Microbacterium*	Y	N
OTN167-C2	Va1	V1–V9	1,442	*Variovorax*	N	N
OTN167-C9	Ps7	V1–V9	1,431	*Pseudomonas*	Y	Y
OTN184-C8	Ba2	V1–V9	1,454	*Bacillus*	Y	Y
OTN189-C6	Ps8	V1–V9	1,428	*Pseudomonas*	Y	Y

^*a*^ Known insecticidal relatives (KIRs) within the genus are indicated along with information on whether any of the insecticidal relatives are known to be mosquitocidal (mKIR); presence in literature for both denoted either Y (yes) or N (no). KIRs were identified based on species within the genus that generated insecticidal activity as a culture or which produced specific insecticidal molecules. Insecticidal activity of most secondary metabolites is concentration dependent; therefore, the simple production of such compounds did not infer activity for confirmatory inclusion in the table.

^*b*^ Denotes *Exiguobacterium* genus due to singular report *Exiguobacterium* spp. exposure significantly affecting *Aedes aegypti* growth and reproductive fitness (26).

### Screening for metabolite-derived insecticidal activity

**Fig 3:**
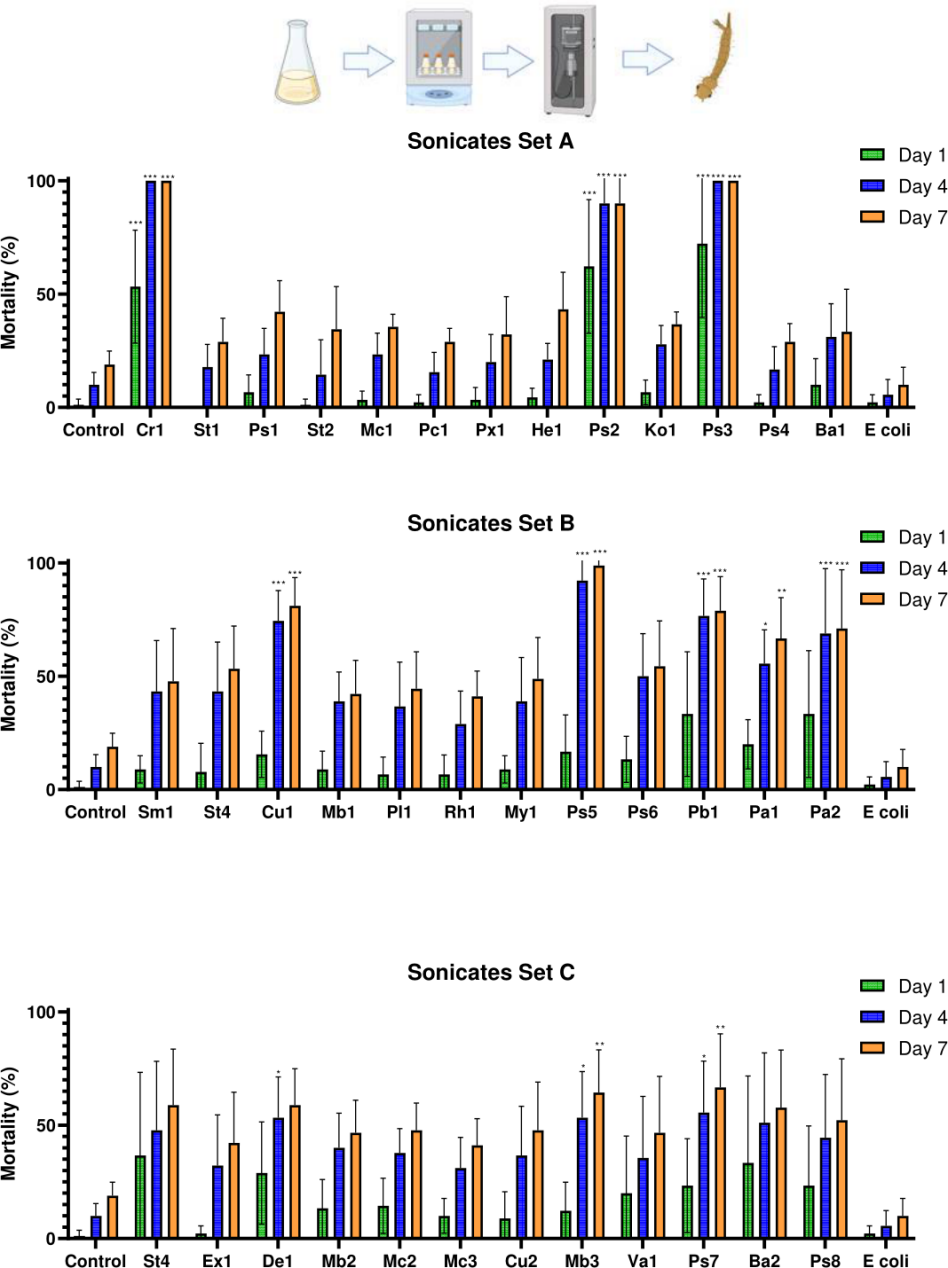
Mortality of *Culex pipiens molestus* larvae exposed to bacterial sonicates of the top 37 “hit” identified in the initial live-culture screening. Controls consisted of a sonicated LB medium dilution and a sonicated culture of *E. coli*. Data are presented as mean mortality within the replicate data at each timepoint. Errors shown are 95% CI. Statistical significance levels (derived from Tukey’s post hoc analyses) as compared to LB controls are denoted as follows: *P < 0.05, **P < 0.01, ***P < 0.001. For Fig. 3 through 7, the labels on the bar graphs represent the suffixes of the names for the individual isolates. Genus key: Cr – *Chryseobacterium*, St – *Stenotrophomonas*, Ps – *Pseudomonas*, Mc – *Micrococcus*, Pc – *Paracoccus*, Px – *Pseudoxanthomonas*, Hb – *Herbiconiux*, Ko – *Kocuria*, Ba – *Bacillus*, Sm – *Streptomyces*, Cu – *Curtobacterium*, Mb – *Microbacterium*, Pl – *Planococcus*, Rh – *Rhodococcus*, My – *Mycobacterium*, Pb – *Phaenarthrobacter*, Pa – *Pantoea*, Ex – *Exiguobacterium*, De – *Dermacoccus*, Va – *Variovorax*. Explanatory figure created in BioRender. Vontas, J. (2025) https://BioRender.com/7n4814z

To assess whether the larvicidal activity we observed was the result of infection by live bacteria or resulted from metabolically-synthesized insecticidal factors, we inactivated liquid LB cultures through sonication-mediated lysis prior to conducting the larvicidal bioassays. This approach enabled the killing of the bacteria and the release of the insecticidal factors into the culture supernatant without compromising their integrity. The sonicated cultures were then tested in larvicidal bioassays at a 1/10 dilution in water. Significant differences between treatments and controls were evident throughout the experiment (*F*_(38,312)_ = 5.355, *P* < 0.001). By 1 day post-exposure (dpe), notable differences in mean mortality were observed between the negative controls and several sonicated bacterial cultures (Fig. 3), with no significant difference between the LB control and sonicated *Escherichia coli* control (*P* = 1.000). Among the sonicated isolates, three caused 50–100% mortality within some replicates inside of 1 day: *Chryseobacterium* OTN1-C11_Cr1 (*P* = 0.001 and *P* = 0.002 vs the LB and *E. coli* controls, respectively), *Pseudomonas* OTN72-C2_Ps2 (*P* < 0.001 in both cases), and *Pseudomonas* OTN78-C5_Ps3 (*P* < 0.001 in both cases). Both *Pseudomonas* OTN72-C2_Ps2 and *Pseudomonas* OTN78-C5_Ps3 produced a significantly higher mean mortality than did *Chryseobacterium* OTN1-C11_Cr1 (*P* < 0.001 in both cases). Although there was no significant difference between the mortality levels caused by *Pseudomonas* OTN72-C2_Ps2 and *Pseudomonas* OTN78-C5_Ps3 (*P* = 1.000).

Mortality at 4 dpe demonstrated clear differences between mortality-inducing treatments and the LB and *E. coli* controls (*F*_(38,312)_ = 9.962, *P* < 0.001). Whereas both negative controls caused little mortality, and no significant differences were found between them (*P* = 1.000), mortality in all replicates reached 100% for isolates *Chryseobacterium* OTN1-C11_Cr1 and *Pseudomonas* OTN78-C5_Ps3, while also reaching near maximum for *Pseudomonas* OTN72-C2_Ps2, except in one outlier repeat, and even in this case, the mortality was significantly greater than for either the LB or *E. coli* control (*P* < 0.001 in all cases). Isolates *Curtobacterium* OTN85-C3_Cu1, *Pseudomonas* OTN111-C7_Ps5, *Paenarthrobacter* OTN131-CD2_Pb1, and *Pantoea* OTN141-C1_Pa1 also achieved their maximum significance in mortality difference after 4 days when compared to either the LB control (*P* < 0.001 in all cases) or the *E. coli* control (*P* < 0.001 in all cases). Isolates *Pantoea* OTN134-CD2_Pa2 (*P* = 0.018), *Dermacoccus* OTN144-C3_De1 (*P* = 0.038), *Microbacterium* OTN166-C1_Mb1 (*P* = 0.038), and *Pseudomonas* OTN167-C9_Ps7 (*P* = 0.018) also caused significant larval mortality when compared to the LB-only control, but, interestingly, at this point there were no significant differences between these four isolates and the *E. coli* control (*P* = 0.054, 0.103, 0.103, and 0.054, respectively).

At 7 dpe, patterns of insecticidal activity among the isolates remained similar. Four of the isolates, *Chryseobacterium* OTN1-C11_Cr1, *Pseudomonas* OTN72-C2_Ps2, *Pseudomonas* OTN78-C5_Ps3, and *Pseudomonas* OTN111-C7_Ps5, exhibited a mean mortality of >90% throughout the entire time course (*P* < 0.001 in all cases). Also, *Curtobacterium* OTN85-C3_Cu1 (*P* < 0.001), *Paenarthrobacter* OTN131-CD2_Pb1 (*P* < 0.001), *Pantoea* OTN134-CD2_Pa1 (*P* = 0.003), *Pantoea* OTN141-C1_Pa2 (*P* < 0.001), *Microbacterium* OTN166-C1_Mb1 (*P* = 0.008), and OTN167-C9_Ps7 (*P* = 0.003) demonstrated significantly greater mortality than did the controls. No significant differences were detected between the LB-only and the *E. coli* controls at the end of the experiment (*P* = 1.000).

### Determination of secretion status, polarity, and killing rate of bacteria-derived insecticidal factors

To further investigate the nature of the bacteria-produced larvicidal factors and determine whether they may be secreted, we conducted mortality assays using extracts from both intact (extracellular) and sonicated (whole culture) bacterial cultures, employing both polar and non-polar solvents. Ethyl acetate was selected as a polar solvent with a very low miscibility in water and ability to gently extract a broad range of polar molecules. Hexane was used as a non-polar solvent for effective extraction of various non-polar molecules. For both solvents, we performed two types of extractions: an extracellular extraction using intact bacteria in their spent culture medium to capture compounds secreted into the medium, and an extraction of sonicated whole cultures that would contain both secreted and whole culture factors. For these assays, a modified culture time was determined based upon a separate experiment (Supplementary File, Part 2) whereby insecticidal efficacy, variable culture times, and cell count data were correlated (Fig. S2). It was found that isolates *Chryseobacterium* OTN1-C11_Cr1, *Pseudomonas* OTN72-C2_Ps2, and Pseudomonas OTN78-C5_Ps3 were able to kill 100% of the larvae within 24 h with between 2 and 4 days culture (Fig. S3 through S5), after which insecticidal efficacy dropped in some cases. Meanwhile, the less efficacious isolates *Bacillus* OTN82-C2_Ba1 (Fig. S6) and *Stenotrophomonas* OTN84-CD3_St4 (Fig. S7) required 3 or more days to cause higher degrees of mortality.

#### Bacterial isolates producing fast-acting non-polar larvicidal factors

**Fig 4:**
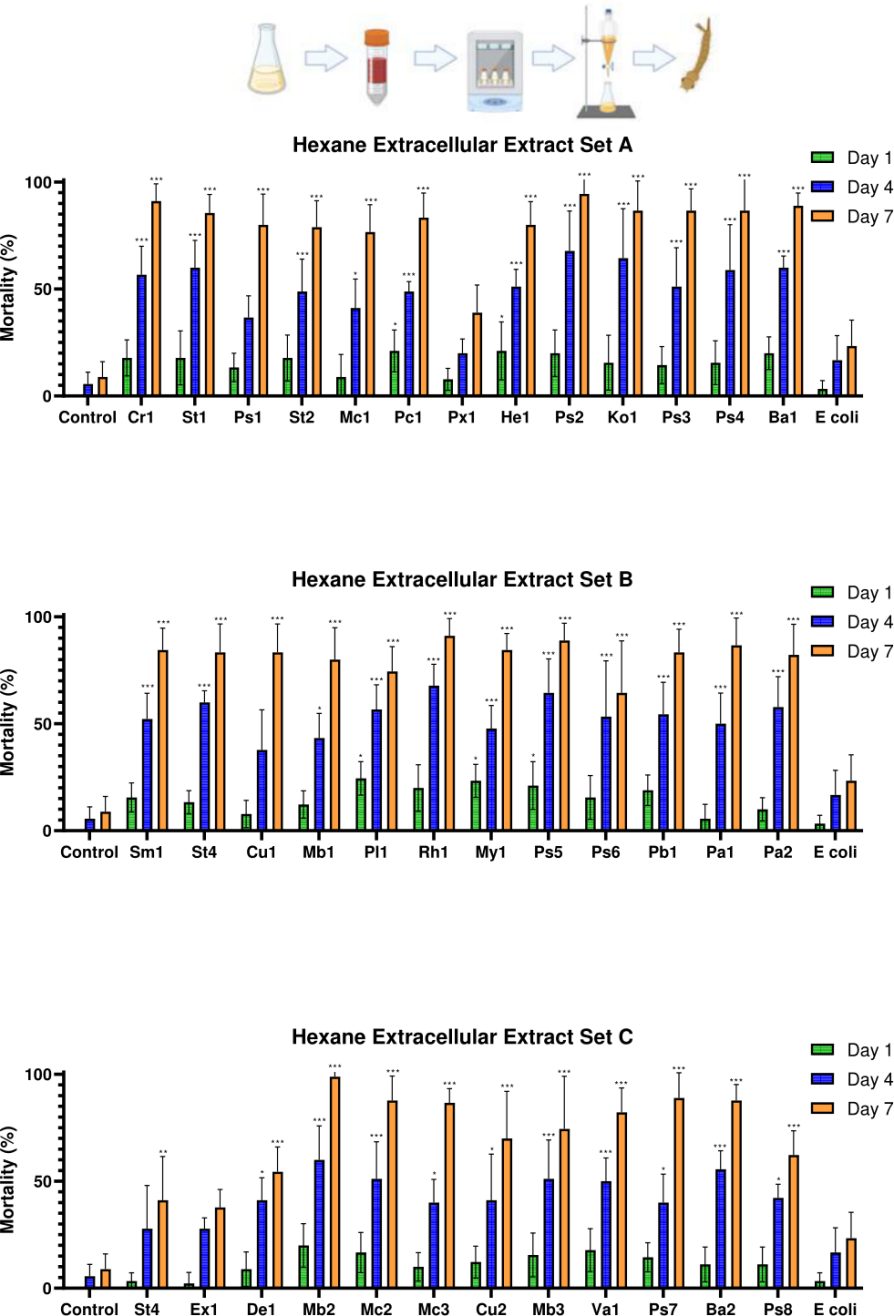
Mortality of *Culex pipiens molestus* larvae exposed to extracellular hexane-extracted bacterial metabolites of the top 37 hits identified in the initial screening. The figure shows the mean mortality at 1, 4, and 7 dpe. Controls consisted of an extracted LB dilution only and an extracellular hexane extract of *E. coli*. Errors shown are 95% CI. Statistical significance levels (derived from Tukey’s post hoc analyses) as compared to LB controls are denoted as follows: *P < 0.05, **P < 0.01, ***P < 0.001. Genus key: Cr – *Chryseobacterium*, St – *Stenotrophomonas*, Ps – *Pseudomonas*, Mc – *Micrococcus*, Pc – *Paracoccus*, Px – *Pseudoxanthomonas*, Hb – *Herbiconiux*, Ko – *Kocuria*, Ba – *Bacillus*, Sm – *Streptomyces*, Cu – *Curtobacterium*, Mb – *Microbacterium*, Pl – *Planococcus*, Rh – *Rhodococcus*, My – *Mycobacterium*, Pb – *Paenarthrobacter*, Pa – *Pantoea*, Ex – *Exiguobacterium*, De – *Dermacoccus*, Va – *Variovorax*. Explanatory cartoon created in BioRender. Vontas, J. (2025) https://BioRender.com/hzuke3o

Already at 1 dpe, significant differences in mortality among treatment groups in the extracellular hexane extract bioassays were observed for a few isolates (*F*_(38,312)_ = 2.681, *P* < 0.001) (Fig. 4). Specifically, isolates *Paracoccus* OTN30-CD7_Pc1 (*P* = 0.036), *Herbiconiux* OTN63-C5_He1 (*P* = 0.036), *Planococcus* OTN85-CD2_Pl1 (*P* = 0.003), *Mycobacterium* OTN110-CD3_My1 (*P* = 0.007), and *Pseudomonas* OTN111-C7_Ps5 (*P* = 0.036) showed significant insecticidal effects. Similarly, significant differences were observed between treatments in the whole culture hexane extract experiments at 1 dpe (*F*_(38,312)_ = 7.496, *P* < 0.001). Post hoc analysis identified *Pseudomonas* OTN72-C2_Ps2 (*P* < 0.001), *Pseudomonas* OTN78-C5_Ps3 (*P* < 0.001), and *Pantoea* OTN134-CD2_Pa2 (*P* = 0.02) as the isolates with significant mortality effects when compared to evaporated hexane controls. Also, *Chryseobacterium* OTN1-C11_Cr1 (*P* = 0.016) and *Paenarthrobacter* OTN131-CD2_Pb1 (*P* = 0.016) demonstrated significantly higher larvicidal activity than did the *E. coli* extract controls.

#### Bacterial isolates producing non-polar larvicidal factors that kill larvae at 4 dpe

At 4 dpe, most extracellular hexane extracts caused significantly higher mortality than either the corresponding hexane or *E. coli* extract controls. The isolates causing the most significant mortality when compared to the hexane extract controls (*P* < 0.001 in all cases) were *Chryseobacterium* OTN1-C11_Cr1, *Pseudomonas* OTN72-C2_Ps2, *Pseudomonas* OTN78-C5_Ps3, and *Pseudomonas* OTN111-C7_Ps5. These isolates also exhibited the strongest insecticidal activity in the initial bioassays of sonicated cultures (see “Screening for metabolite-derived insecticidal activity,” above), being significantly different from the *E. coli* controls (*P* = 0.004, *P* < 0.001, *P* = 0.043, and *P* < 0.001, respectively). No significant differences were observed between the hexane and *E. coli* control groups (*P* = 1.000). At 4 dpe, the whole culture hexane extracts of isolates *Chryseobacterium* OTN1-C11_Cr1, *Pseudomonas* OTN72-C2_Ps2, and *Pseudomonas* OTN78-C5_Ps3 continued to show highly significant mortality (*P* < 0.001), consistent with the extracellular hexane extract trials. However, *Pseudomonas* OTN111-C7_Ps5 did not show significant mortality when compared to the evaporated hexane control (*P* = 0.976), indicating some variability in its effectiveness. Overall, 16 of the 37 whole culture hexane extracts induced significant mortality when compared to both the evaporated hexane and *E. coli* hexane extract controls at 4 dpe.

#### Larvicidal activity of bacteria-produced non-polar factors at 7 dpe

**Fig 5:**
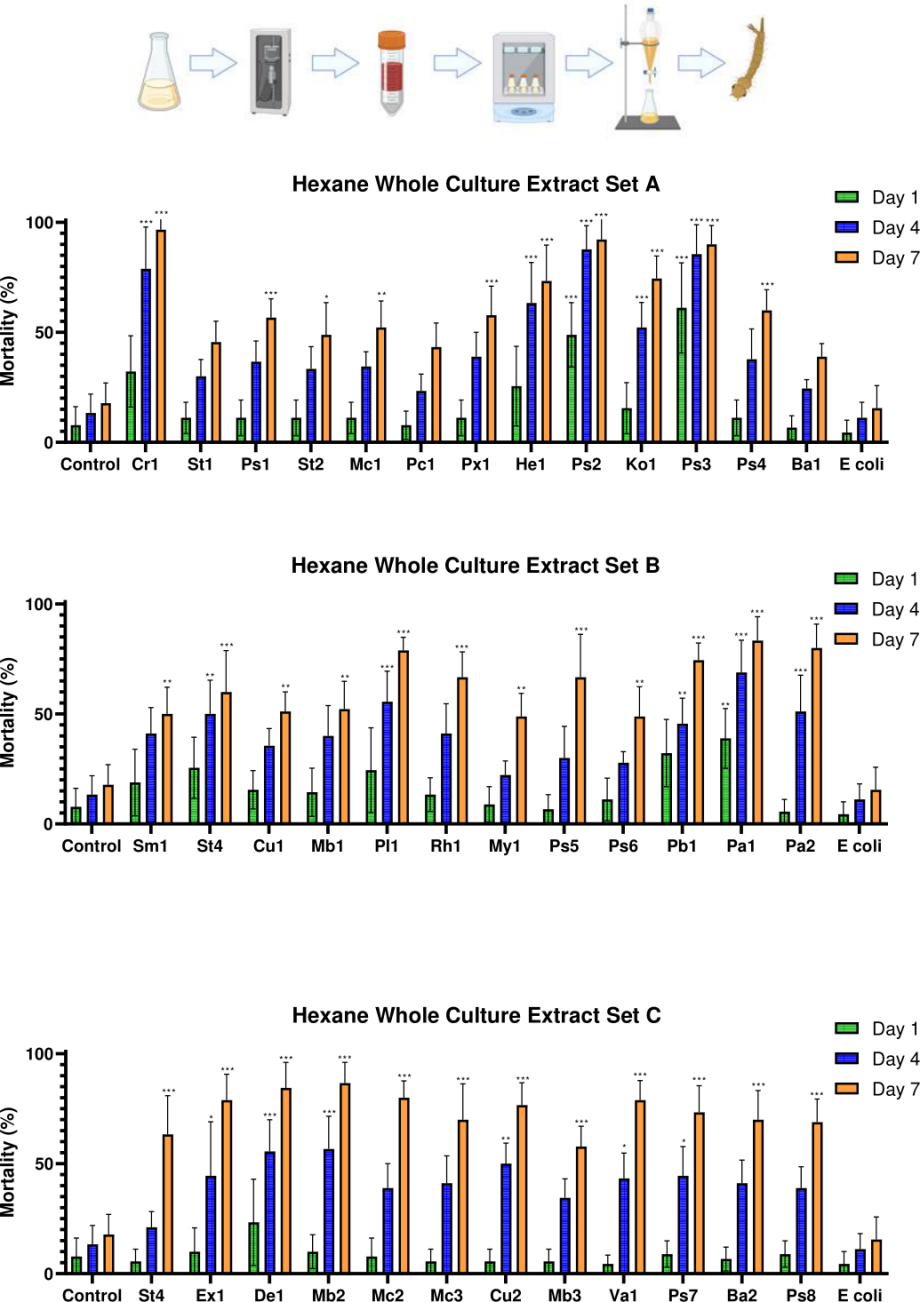
Mortality of *Culex pipiens molestus* larvae exposed to whole culture hexane-extracted bacterial metabolites of the top 37 hits identified in the initial screening. The figure shows the mean mortality at 1, 4, and 7 dpe. Controls consisted of an extracted LB dilution only and a whole culture hexane extract of *E. coli*. Errors shown are 95% CI. Statistical significance levels (derived from Tukey’s post hoc analyses) as compared to LB controls are denoted as follows: *P < 0.05, **P < 0.01, ***P < 0.001. Genus key: Cr – *Chryseobacterium*, St – *Stenotrophomonas*, Ps – *Pseudomonas*, Mc – *Micrococcus*, Pc – *Paracoccus*, Px – *Pseudoxanthomonas*, Hb – *Herbiconiux*, Ko – *Kocuria*, Ba – *Bacillus*, Sm – *Streptomyces*, Cu – *Curtobacterium*, Mb – *Microbacterium*, Pl – *Planococcus*, Rh – *Rhodococcus*, My – *Mycobacterium*, Pb – *Paenarthrobacter*, Pa – *Pantoea*, Ex – *Exiguobacterium*, De – *Dermacoccus*, Va – *Variovorax*. Explanatory cartoon created in BioRender. Vontas, J. (2025) https://BioRender.com/n64z815

At the final time point of 7 dpe, similar larvicidal potencies were observed, with most bacterial extracellular hexane extracts causing significantly higher mortality than the *E. coli* hexane extract control. ANOVA analysis indicated considerable variation across treatments in the extracellular hexane extract studies (*F*_(38,312)_ = 12.396, *P* < 0.001). Four isolates with the strongest and most consistent larvicidal activity in the initial sonicated culture screen—*Chryseobacterium* OTN1-C11_Cr1, *Pseudomonas* OTN72-C2_Ps2, *Pseudomonas* OTN78-C5_Ps3, and *Pseudomonas* OTN111-C7_Ps5—caused significant mortality when extracted into hexane, as compared to both the evaporated hexane control and the extracellular *E. coli* hexane extract at 7 dpe (*P* < 0.001 in all cases). No significant differences were found between the control hexane and *E. coli* hexane extracts (*P* = 0.999). The whole culture hexane extracts yielded similar results (Fig. 5), with significant differences among treatment groups (*F*_(38,312)_ = 13.457, *P* < 0.001). Most isolates caused significantly higher mortality than did either the evaporated hexane or *E. coli* hexane extract control. Isolates *Stenotrophomonas* OTN3-C1, *Paracoccus* OTN30-CD7_Pc1, and *Streptomyces* OTN82-C2_Ba1 did not cause statistically significant mortality when compared to the evaporated hexane control (*P* = 0.055, *P* = 0.141, and *P* = 0.547, respectively), but all other isolates demonstrated significant mortality (*P* < 0.05 for all). Consistent with the extracellular hexane extract assays, *Chryseobacterium* OTN1-C11_Cr1, *Pseudomonas* OTN72-C2_Ps2, *Pseudomonas* OTN78-C5_Ps3, and *Pseudomonas* OTN111-C7_Ps4 caused highly significant mortality (*P* < 0.001), and no differences were detected between the LB and *E. coli* controls (*P* = 1.000).

#### Bacterial isolates producing fast-acting polar larvicidal factors

While no significant mortality differences were found between any treatment groups in the extracellular ethyl acetate extract bioassays at 1 dpe (*F*_(38,312)_ = 1.241, *P* = 0.165), several whole culture ethyl acetate extractions showed overall significance between treatments at 1 dpe (*F*_(38,312)_ = 8.063, *P* < 0.001). Notably, isolates *Pseudomonas* OTN115-C6_Ps6 and *Pantoea* OTN134-CD2_Pa2 produced significant mortality when compared to both evaporated ethyl acetate and *E. coli* extract controls (*P* < 0.001 in all cases).

#### Larvicidal activity of bacteria-produced polar factors at 4 dpe

**Fig 6:**
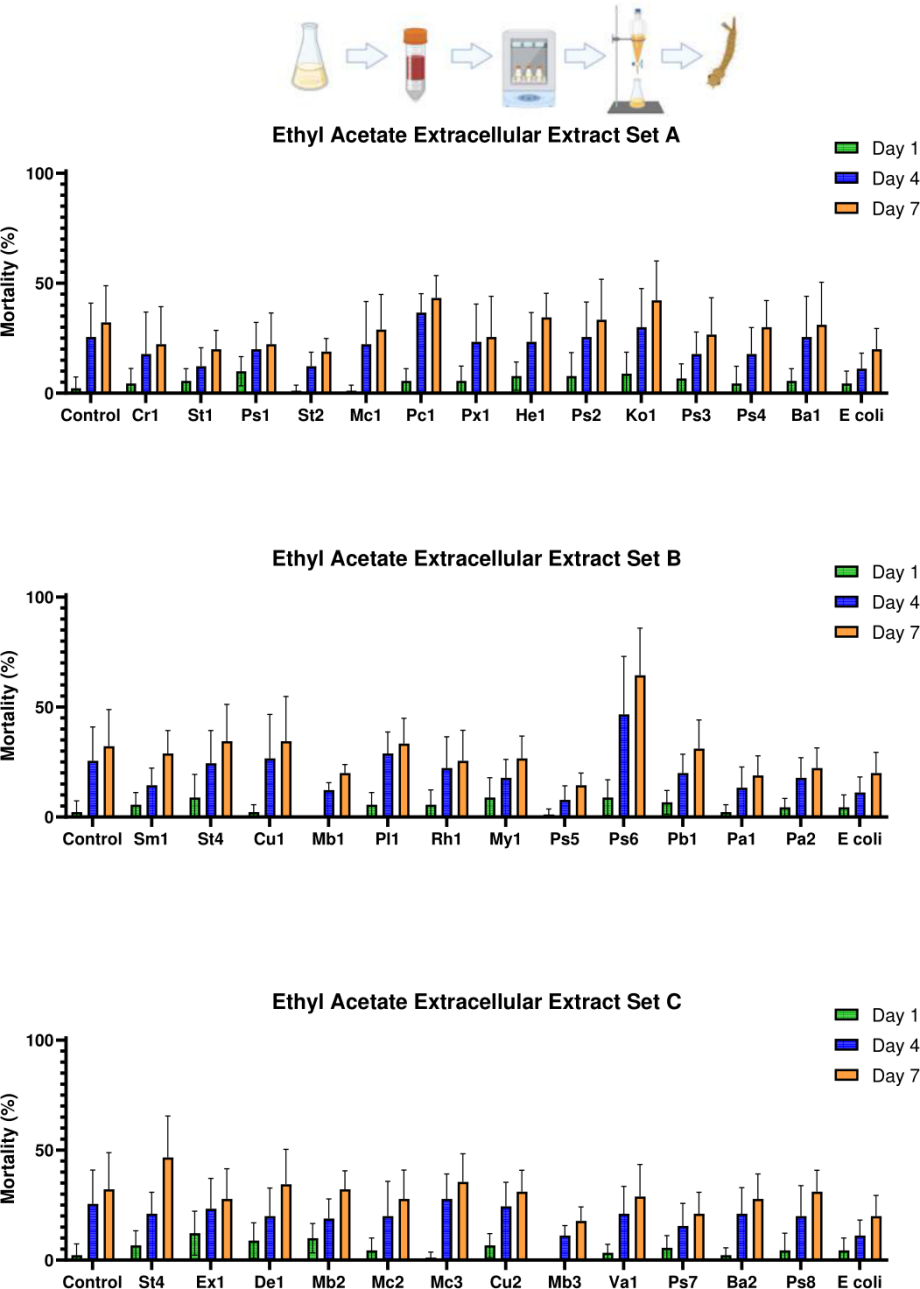
Mortality of *Culex pipiens molestus* larvae exposed to extracellular ethyl acetate-extracted bacterial metabolites of the top 37 hits identified in the initial screening. The figure shows the mean mortality at 1, 4, and 7 dpe. Controls consisted of an extracted LB dilution only and an extracellular ethyl acetate extract of *E. coli*. Errors shown are 95% CI. Statistical significance levels (derived from Tukey’s post hoc analyses) as compared to LB controls are denoted as follows: *P < 0.05, **P < 0.01, ***P < 0.001. Genus key: Cr – *Chryseobacterium*, St – *Stenotrophomonas*, Ps – *Pseudomonas*, Mc – *Micrococcus*, Pc – *Paracoccus*, Px – *Pseudoxanthomonas*, Hb – *Herbiconiux*, Ko – *Kocuria*, Ba – *Bacillus*, Sm – *Streptomyces*, Cu – *Curtobacterium*, Mb – *Microbacterium*, Pl – *Planococcus*, Rh – *Rhodococcus*, My – *Mycobacterium*, Pb – *Paenarthrobacter*, Pa – *Pantoea*, Ex – *Exiguobacterium*, De – *Dermacoccus*, Va – *Variovorax*. Explanatory cartoon created in BioRender. Vontas, J. (2025) https://BioRender.com/hzuke3o

**Fig 7:**
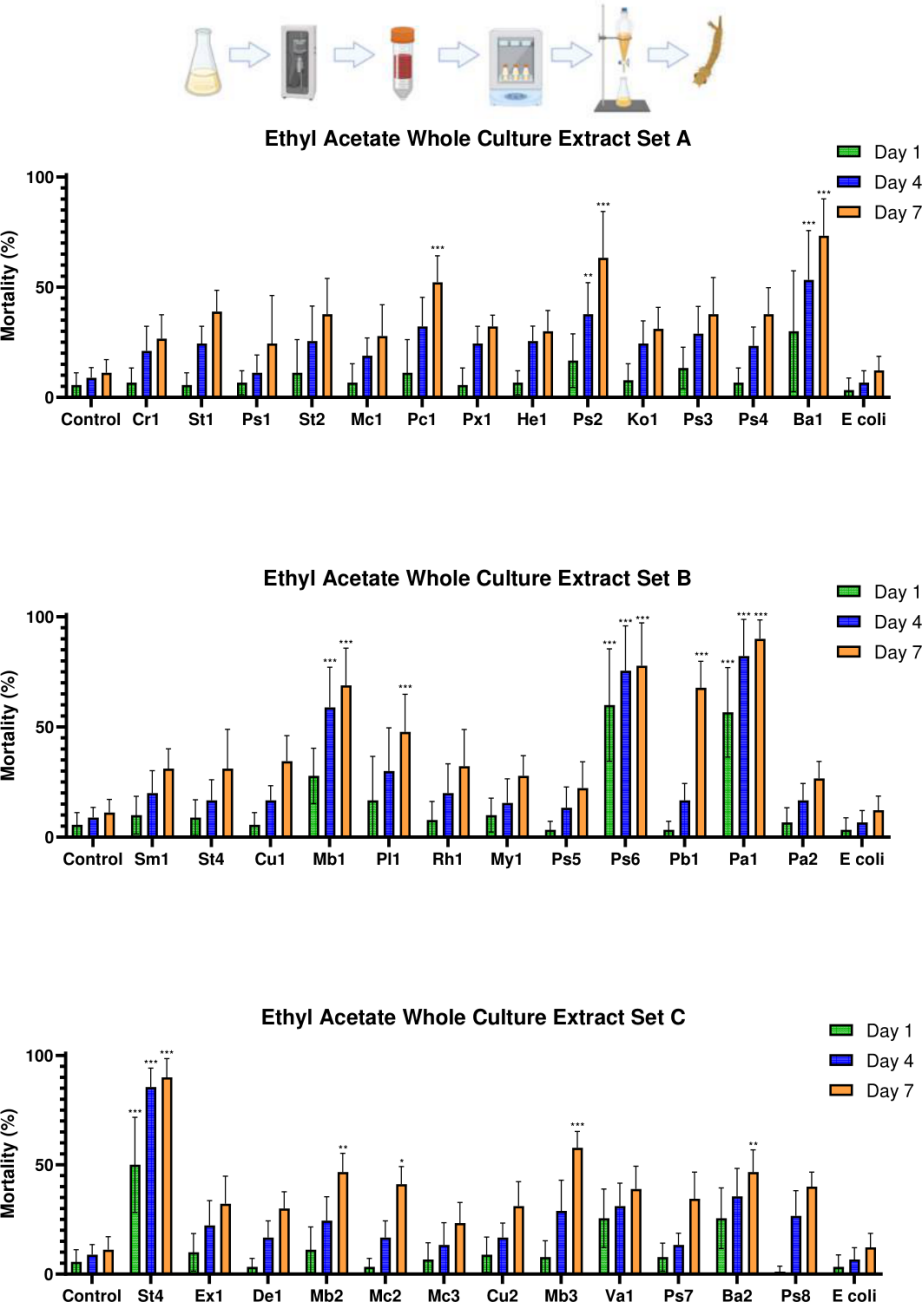
Mortality of *Culex pipiens molestus* larvae exposed to whole culture ethyl acetate-extracted bacterial metabolites of the top 37 hits identified in the initial screening. The figure shows the mean mortality at 1, 4, and 7 dpe. Controls consisted of an extracted LB dilution only and a whole culture ethyl acetate extract of *E. coli*. Errors shown are 95% CI. Statistical significance levels (derived from Tukey’s post hoc analyses) as compared to LB controls are denoted as follows: *P < 0.05, **P < 0.01, ***P < 0.001. Genus key: Cr – *Chryseobacterium*, St – *Stenotrophomonas*, Ps – *Pseudomonas*, Mc – *Micrococcus*, Pc – *Paracoccus*, Px – *Pseudoxanthomonas*, Hb – *Herbiconiux*, Ko – *Kocuria*, Ba – *Bacillus*, Sm – *Streptomyces*, Cu – *Curtobacterium*, Mb – *Microbacterium*, Pl – *Planococcus*, Rh – *Rhodococcus*, My – *Mycobacterium*, Pb – *Paenarthrobacter*, Pa – *Pantoea*, Ex – *Exuigobacterium*, De – *Dermacoccus*, Va – *Variovorax*. Explanatory cartoon created in BioRender. Vontas, J. (2025) https://BioRender.com/n64z815

Only the extracellular ethyl acetate extract of isolate *Pseudomonas* OTN115-C6_Ps6 demonstrated significantly higher mortality than did the *E. coli* extracellular ethyl acetate extract control (*P* = 0.007) at 4 dpe, with no significant difference when compared to the ethyl acetate extract control (*P* = 0.800) (Fig. 6). No significant difference was observed between the extracellular *E. coli* ethyl acetate extract control and the ethyl acetate extract control (*P* = 0.999) at 4 dpe. In contrast, whole culture extracts from isolates *Streptomyces* OTN82-C2_Ba1, *Microbacterium* OTN85-C8_Mb1, *Pseudomonas* OTN115-C6_Ps6, *Pantoea* OTN134-CD2_Pa2, and *Stenotrophomonas* OTN141-CD4_St4 showed greater variability in their larvicidal effects (*F*_(38,312)_ = 13.838, *P* < 0.001), with several isolates causing significant mortality relative to controls at 4 dpe (Fig. 7). Specifically, isolates *Bacillus* OTN82-C2_Ba1, *Microbacterium* OTN85-C8_Mb1, *Pseudomonas* OTN115-C6_Ps6, *Pantoea* OTN134-CD2_Pa2, and *Stenotrophomonas* OTN141-CD4_St4 all exhibited highly significant larvicidal activities when compared to both control groups (*P* < 0.001). In addition, *Pseudomonas* OTN72-C2_Ps2 showed significantly greater mortality than did the evaporated ethyl acetate extract control (*P* = 0.033) and the *E. coli* extract control (*P* = 0.01).

#### Larvicidal activity of bacteria-produced polar factors at 7 dpe

Bioassays with the extracellular ethyl acetate extracts revealed that larval mortality at 7 dpe was similar to that observed at 4 dpe; only isolate *Pseudomonas* OTN115-C6_Ps6 caused significantly higher mortality when compared to the extracellular *E. coli* ethyl acetate extract control (*P* < 0.001), but showed no significant difference from the evaporated ethyl acetate control (*P* = 0.059). No significant difference was found between the evaporated ethyl acetate and the extracellular *E. coli* extract controls (*P* = 1.000). In contrast, whole culture ethyl acetate extracts showed notable improvements in efficacy and substantial variability among treatments (*F*_(38,312)_ = 12.603, *P* < 0.001). Isolates *Paracoccus* OTN30-CD7_Pc1, *Pseudomonas* OTN72-C2_Ps2, *Streptomyces* OTN82-C2_Ba1, *Streptomyces* OTN85-C8_Mb1, *Pseudomonas* OTN115-C6_Ps6, *Paenarthrobacter* OTN131-CD2_Pb1, *Pantoea* OTN134-CD2_Pa2, *Stenotrophomonas* OTN141-CD4_St4, and *Microbacterium* OTN166-C1_Mb3 all caused highly significant mortality when compared to both the evaporated ethyl acetate and the whole culture *E. coli* ethyl acetate extract controls (*P* < 0.001 in all cases). This trial also demonstrated the highest range of efficacy differences among treatments (Fig. 7).

## DISCUSSION

**Fig 8:**
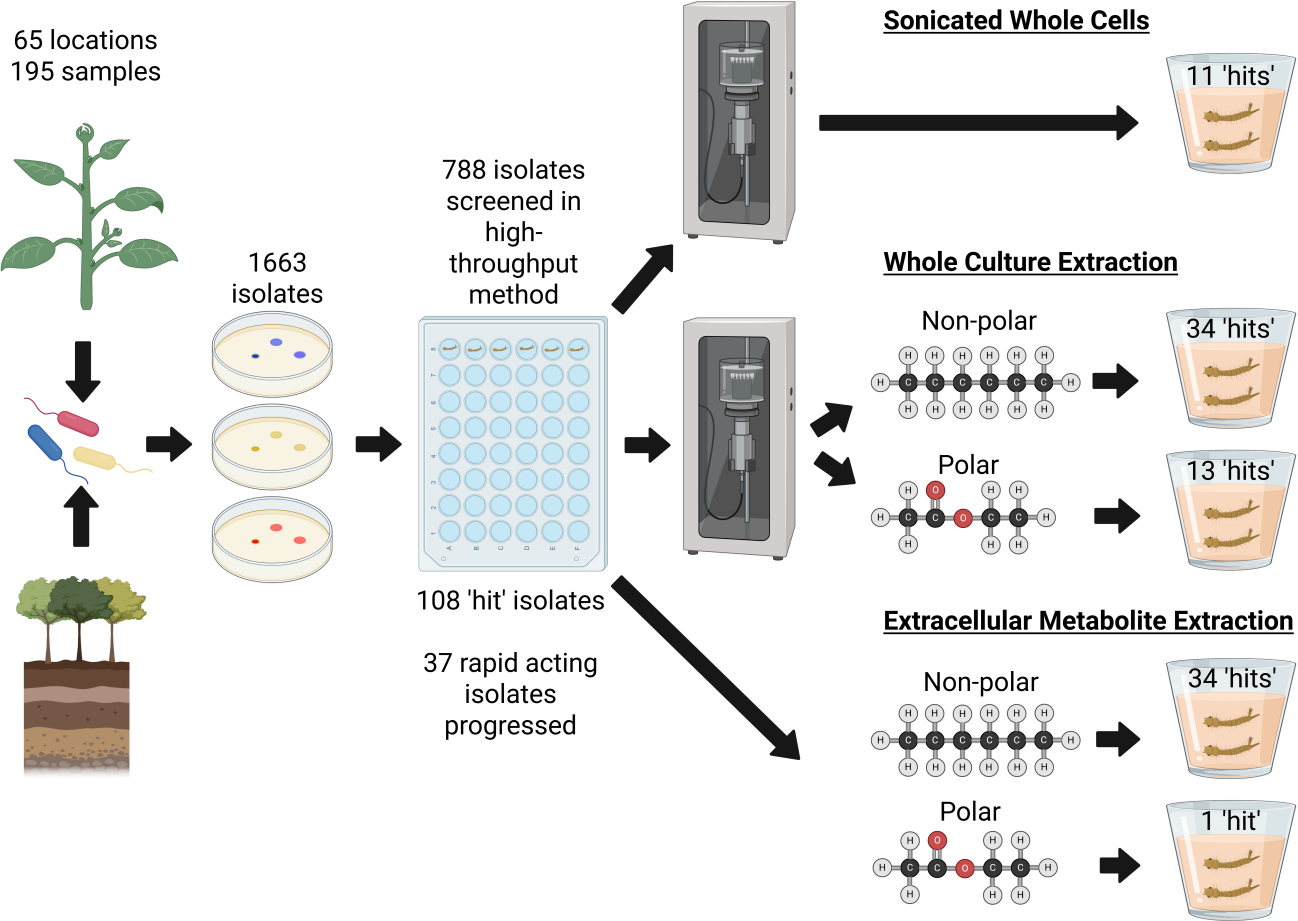
Flowchart outlining the pipeline designed for discovery of insecticidal bacteria and predictions of metabolite properties. Figure created in BioRender. Vontas, J. (2025) https://BioRender.com/bv458q8

Here, we have performed the first extensive survey of bacterial isolates from various environments across the Mediterranean island of Crete, to identify bacteria that produce abiotic larvicidal factors with the potential to develop the next generation of sustainable biopesticides (Fig. 8). Out of 788 bacterial isolates tested for larvicidal activity as live cultures, more than 10% (*n* = 108) showed insecticidal properties in the preliminary screening, with 37 isolates demonstrating high efficacy as rapid-acting larvicides against *Cx. pipiens molestus* larvae. These 37 isolates represent 20 genera, many of which were not previously recognized as having insecticidal effects. No trends were observed between the habitat from which isolates were collected and their likelihood of insecticidal action. This is most likely due to the sampling choices, where we specifically and predominantly targeted microbial habitats that are known to harbor insecticidal bacteria, or that may interact ecologically with the insects. A random sampling approach may have revealed such relationships, albeit at the cost of a significantly greater insecticidal-isolate redundancy and labour.

Further screening revealed that the insecticidal actions of the 37 most rapid-acting bacteria were mostly mediated by bacterially produced factors, such as proteins and secondary metabolites, rather than infection by live bacteria. Also, extracellular non-polar metabolites were generally the predominant insecticidal factors; in contrast, extraction of insecticidal polar compounds required cell lysis in all but one case to allow effective access to the active molecules.

Among the top 37 insecticidal isolates, the majority (*n* = 25, 68%) were found either in the plant rhizosphere (*n* = 11, 29.73%) or as endophytes within plant tissues (*n* = 14, 37.84%). Although the occurrence of endophytic fungi (27), including entomopathogenic-endophytic fungi such as *Metarhizium* spp. (28) and endophytic bacteria (29) within plant tissues are well known, but their role in plant protection as insect deterrents and natural pesticides has only recently been explored (30). It is now recognized that plants actively recruit microbiota in the rhizosphere in response to insect attacks (22), highlighting the success of the strategic focus on rhizosphere microbes in this study’s discovery phase, and underscoring the significance of these environments in the rapidly advancing field of insecticidal microbe discovery.

Several *Chryseobacterium* species are recognized as common gut microbiota in mosquitoes (31), and they are thought to play significant roles in blood digestion processes. In this study, the *Chryseobacterium* isolate showed high larvicidal efficacy, underscoring the diversity of secreted metabolites across closely related species within the genus. This finding highlights the importance of thoroughly screening microbiota for insecticidal activity, regardless of taxonomic similarity. Notably, one species, *Chryseobacterium arthrosphaerae*, produces insecticidal proteins that target corn rootworm (*Diabrotica virgifera*) (32). Although it is possible that a similar protein is produced by the isolate *Chryseobacterium* OTN1-C11_Cr1, solvent extraction trials suggested that non-polar metabolites, rather than typically polar proteins, are primarily responsible for the insecticidal activity observed with this isolate.

In contrast, the genus *Pseudomonas* is well documented for its broad spectrum of insecticidal compounds active against many insects including mosquitoes (33–38). Although some active compounds have been identified, such as the aphicidal Orfamide A compound produced by *Pseudomonas protegens* (39), many remain to be fully characterized. This particular species - *Pseudomonas protegens* - closely related to isolate *Pseudomonas* OTN78-C5_Ps3, is also an effective larvicide against *Cx. pipiens* (40); however, the specific mosquitocidal compound remains unknown for both OTN78-C5_Ps3 and *P. protegens*. Given that ethyl acetate extraction did not yield significant activity, it is possible that a different molecule may be responsible in our case.

The extracellular hexane extracts—more representative of secreted non-polar metabolites—were slightly more likely to show larvicidal activity than were the whole culture extracts. This difference may be the result of the release of hydrophobic, non-insecticidal compounds during cell lysis, which could reduce extraction efficiency. Conversely, whole culture ethyl acetate extracts were more effective, potentially because of an increased presence of polar compounds, including proteins and peptides, which are more abundant inside cells. These differences in extract activity provide insights into the chemical nature of insecticidal compounds and can guide future isolation and formulation efforts.

Our study also revealed variation in the speed of larval mortality caused by different isolates, an important factor for developing effective insecticides. For example, the slow-acting mortality of *Pseudomonas* OTN111-C7_Ps5 may involve its accumulation in mosquito larvae over time. Such slow-acting compounds could be valuable for longer-lasting insecticidal effects, although they raise concerns about potential bioaccumulation in non-target species, similar to the effects of compounds such as DDT (41) and diclofenac (42, 43). A thorough characterization of these compounds and their modes of action will be critical.

Other isolates demonstrated rapid action, inducing high mortality within the first few days, but no significant increase over extended periods. For example, *Curtobacterium* OTN85-C3_Cu1 achieved an 81.1% mortality rate within 4 days, with no further increase by day 7, suggesting the involvement of rapidly acting, potentially degradable products like peptides, which can break down in water. Such compounds might be suited to formulations for short-term applications or use in controlled environments, such as sugar-bait traps.

*Pseudomonas* OTN115-C6_Ps6 showed intriguing variability in its larvicidal activity, inducing high mortality in some assays but not in others and hinting at the involvement of a metabolite that requires specific conditions for optimal production.

Importantly, neither hexane nor ethyl acetate extracts of *E. coli* adversely affected larval survival, suggesting that *Cx. pipiens*, which can thrive in polluted waters (44, 45), is highly tolerant to *E. coli* metabolites. In fact, the presence of *E. coli* in mosquito larvae guts has been shown to accelerate growth (46). This finding suggests that a few specific metabolites with insecticidal effects, rather than the general metabolic byproducts of bacteria, may account for the activity observed from selected isolates. Comparing chromatographic profiles across active extracts could help identify these key metabolites and guide future insecticidal development.

## MATERIALS AND METHODS

### Isolate discovery

Soil, water, and plant samples were collected in 15 mL tubes. Non-endophyte and insect samples were vortexed in 1× phosphate-buffered solution (PBS) to disperse the microbial content within the PBS. The organic matter was allowed to settle for 5 min, after which a sterile inoculating loop was used to pick up the supernatant and plate it onto Luria Bertani (LB) agar or a 1:100 dilution of Czapek agar to support the recovery of oligotrophic bacteria.

For the recovery of endophytic microbes, plant tissues were briefly dipped in a 1% bleach solution for 5 s, followed by a 5 s dip in 70% ethanol. A sterile scalpel was used to expose the core of the plant tissue, from which samples were either streaked directly onto agar or mounted onto the plates. The same agar media types were used for both endophytic and non-endophytic samples. Plates were incubated at 27 ± 1°C for 2–7 days until distinct colonies of microbial growth were visible.

Isolates were then subcultured onto fresh LB agar plates to ensure purity, and once contamination-free cultures were obtained, each isolate was assigned a unique library code. Detailed photographic documentation and morphological descriptions were prepared for each isolate.

### Microbial library generation

A cryo-preserved microbial isolate library was created by inoculating single colonies into 5 mL of LB broth in a sterile, capped test tube. The samples were then grown in a shaking incubator at 150 rpm and 27 ± 1°C for up to 3 days, depending on the visible growth of bacteria in the medium. Control tubes containing only LB broth were included in the incubation process to monitor for any potential contamination, and aliquots from each test tube were plated onto LB agar as needed to assess purity. Following the growth period, 500 µL of bacterial culture were transferred into a 1.5 mL Eppendorf tube containing 500 µL of 50% glycerol under sterile conditions. Duplicate Eppendorf tubes were prepared and stored at −80°C.

### Mosquito rearing

The *Culex pipiens molestus* colony was maintained at 27 ± 1°C, 70% relative humidity, with a 16:8 (L) light cycle. Larvae were hatched in tap water and fed with Vipan Natura tropical flaked fish food (Sera, Heinsberg, Germany) throughout their development. Pupae were transferred to fresh distilled water in a 30 × 30 × 30 cm Bugdorm-1 rearing cage (Australian Entomological Supplies, South Murwillumbah, Australia). Once adults emerged, fresh water was provided for autogenic oviposition, and a 10% white cane sugar solution (AB Supermarket, Greece) on cotton wool was offered as a food source. Eggs were collected and transferred to fresh larval containers immediately after oviposition.

### Bacterial culturing

Bacteria were selected from the library based on semi-indicative morphological traits known to correlate with insecticidal properties including: (i) pigmentation (47), (ii) filamentous or swarming growth patterns on agar (48), and (iii) production of noticeable color changes in agar medium, which may indicate strong metabolite production (49). Bacteria were transferred from LB agar plates to sterile LB broth medium using a sterile P200 pipette tip and then incubated in a Witeg IS-20R shaking incubator (Witeg, Wertheim, Germany) at 27 ± 1°C and 150 rpm. For the initial high-throughput screen, a 5 mL liquid culture of each isolate was prepared. These isolates were cultured for 24–48 h, during which time the color change in the medium indicated strong bacterial growth. This preliminary growth phase was followed by intensive screening of the top candidate bacteria.

### Sonication of bacterial cultures

To inactivate the bacteria through sonication, 50 mL cultures were prepared and decanted into 50 mL Falcon tubes and subjected to sonication on ice. The bacteria were sonicated using an intermittent operating cycle of 1 min on and 1.5 min off, at an amplitude of 60% using a ¼ in. microtip probe of a Branson SFX 150 Digital Sonifier (Emerson, Missouri, USA). The total cycle was 25 min, during which time the bacteria were sonicated for 10 min. A sterile inoculation loop was used to inoculate each sonicate onto an LB agar plate for 24 h at 27 ± 1°C to test for the inactivation of live cells.

### Solvent extractions of sonicated bacterial cultures

Bacterial LB broth cultures (100 mL each in sterile 250 mL conical flasks with sterile foil caps) were prepared by incubation in a shaking incubator at 27 ± 1°C and 150 rpm for 3 days. The extended culture time was chosen after cell culture time, insecticidal efficacy, and cell count data were determined for five key strains identified in the sonication screening phase (Supplementary File, Part 2). To each culture, either 50 mL of n-hexane (AR grade, Fisher Scientific, Germany) or 50 mL of ethyl acetate (AR grade, Honeywell, Germany) was added to the surface. The flasks were re-sealed and incubated in the shaking incubator at 10 ± 1°C and 45 rpm for 3 days. Lack of cell lysis was confirmed by light microscopy to ensure that extracted metabolites were extracellularly secreted.

A secondary method was employed to further address whether insecticidal compounds were contained only within the culture medium, or whether higher concentrations of the metabolites could be obtained by lysing the cells to expose intracellular metabolites: A sonication step employing the same methodology as described in “Sonication of bacterial cultures,” above, was included after culturing, prior to the extraction steps.

At the end of the second 3 days of extraction, the broth and organic solvents were decanted into a 500 mL separating funnel, and all the bacterial broth was removed, with the remaining 50 mL of the clear solvent extract being decanted and filtered through Whatman number 4 filter paper into a clean glass beaker. The solvent was evaporated from the sample in a fume hood at 24 ± 1°C before the extract was resuspended for bioassay. When extractions were conducted using hexane, the samples were resuspended in 0.03% Triton X-100 solution to aid the suspension of the non-polar component (50); compounds extracted into ethyl acetate were resuspended in distilled water only, to facilitate the recovery of polar compounds.

### Larvicidal bioassays with live bacterial cultures

For each treatment, 900 µL of distilled water was added to a well in a 48-well plate. A 100 µL aliquot of live bacterial broth (see “Bacterial culturing,” above) was added to 900 µL of distilled water in a 48-well plate together with three third- or fourth-instar mosquito larvae. Controls contained distilled water and sterile LB broth. Mortality recorded at 24 h intervals over 7 days. Two repeats (*n* = 6) were conducted for each bacterial isolate.

### Larvicidal bioassays with sonicated bacterial cultures

Bioassays were conducted using 250 mL tubs (OD: 8 cm) with perforated lids to allow for air exchange. In each assay, 90 mL of distilled water containing a total of 10 third- to fourth-instar *Cx. pipiens molestus* larvae were treated with 10 mL of sonicated broth. Control experiments consisted of a treatment with 90 mL of distilled water and 10 mL of sterile LB broth; a secondary negative control involved 10 mL of an *E. coli* liquid culture grown and sonicated under the same conditions as the novel insecticidal bacteria. A small amount of flaked fish food was added to each experiment to provide additional sustenance. Three technical replicates were conducted for each bacterial preparation of 30 mL, and the whole experiment was conducted a further two times to allow for full biological replication (*n* = 90). Mortality, pupation, and emergence were recorded daily for a total of 7 days, at which point the experiment was terminated.

### Larvicidal bioassays with solvent extracts of sonicated bacterial cultures

Distilled water (90 mL) containing 10 third to fourth-instar *Cx. pipiens molestus* larvae was mixed with 10 mL of bacterial extract, and the larvae were provided with fish food. Controls consisted of evaporated solvent resuspended in water or a 0.03% Triton X-100 solution, as above, in order to rule out the possibility that solvent-stabilizing or surfactant compounds were contributing to the larval mortality. Mortality, pupation, and emergence were recorded every 24 h for a total of 7 days, and the experiment involved a total of three biological replicates, each with nine technical replicates (*n* = 90).

### 16S rRNA sequencing and identification to the genus level

Genomic DNA was subjected to polymerase chain reaction (PCR) using GO-Taq master mix (Promega Co. Madison, USA) according to the manufacturer’s instructions with the primers 27F (5′-AGAGTTTGATYMTGGCTCAG-3′) and 1492R (5′-TACGGYTACCTTGTTACGACTT-3′) (51), and the thermocycler conditions: 95°C for 3 min, 25 cycles of 95°C for 45 s, 50°C for 45 s and 68°C for 90 s, and 68°C for 5 min. PCR products were confirmed on 1% agarose gels and purified using the NucleoSpin Gel and PCR Clean-up Kit (Macherey-Nagel GmbH & Co. KG, Dueren, Germany) for Sanger sequencing using three primers: 27F, 1492R, and 806R (5′- GGACTACHVGGGTWTCTAAT-3′). Raw sequences were edited using BioEdit v.7.7.1, and sequences for each isolate were assembled using MEGA v11.0.13 (52, 53). 16S rRNA sequences were used as queries for BLASTn searches against a manually curated 16S rRNA database from EzBioCloud (54). Sequences were aligned using MAFFT v7.520 (55), and phylogenetic trees were constructed using the top 50 matches from EzBioCloud and the full-length 16S rRNA sequence from *E. coli* (GenBank accession number MN900682.1) as an outgroup (55). Maximum-likelihood trees for each isolate were constructed in IQTree v1.6.12 (56) according to the best-fit model from ModelFinder with 10,000 bootstrap iterations (57). Phylogenetic trees were visualized using FigTree v1.4.4 (available on: http://tree.bio.ed.ac.uk/software/figtree, accessed on 8 February 2024).

### Statistical analyses

Mortality data generated in the secondary screening phase using sonicates and solvent extracts were analysed via one-way ANOVA, with Tukey’s post hoc test employed for multiple comparisons between treatments. All data were analysed in SPSS v.22 (IBM Corporation, USA), and all figures were generated using Prism GraphPad v.8 (GraphPad Software, USA). Mortality data generated in the initial coarse screen were not statistically analysed, but mean survival time was calculated by averaging the cumulative survival time for each individual larva within a treatment group.

## Data Availability

All data relating to bioassays conducted within the study can be found in the Figshare repository at 10.6084/m9.figshare.29107817. Genomic data relating to the 16S regions of the core 37 bacterial isolates can be found at the GenBank repository under the accession numbers PV185360–PV185396.

## SUPPLEMENTAL MATERIAL

Supplemental material aem.00966-25-s0001.docx Figures S1 to S7.

ASM does not own the copyrights to Supplemental Material that may be linked to, or accessed through, an article. The authors have granted ASM a non-exclusive, world-wide license to publish the Supplemental Material files. Please contact the corresponding author directly for reuse.
