# Supplementary material for: Bacteria isolated from biodiverse Mediterranean island habitats yield a large array of biopesticidal metabolites against mosquito larvae
Source: Appl Environ Microbiol. 2025 Jul 7;91(8):e00966-25. doi: 10.1128/aem.00966-25 (PMC12366336; doi:10.1128/aem.00966-25)
Supplement: Supplemental material — Figures S1 to S7. [file aem.00966-25-s0001.docx]

1. **Daily survival of Culex pipiens molestus larvae exposed to live cell cultures of 108 bacteria with insecticidal properties**

The following are time-course survival curves relating to initial coarse-screen data detailed in section 4.7, and the corresponding results presented in section 2.2 of the main article. The experiments consisted of a 10% dilution of live bacterial cells in distilled water, and three *Culex pipiens* larvae exposed to the treatments and monitored over seven days. Mortality was assessed on a daily basis, and control experiments consisted of a 10% Luria Bertani media dilution only. Daily mortalities and survival probability are provided for all 108 identified ‘hit’ isolates, those isolates listed in the key for each graph that have an asterisk (*) were shown to be the most rapid acting and selected as the 37 ‘top hits’ for detailed assay (table 1 in the main article).

**Supplementary Figure S1 Panel A:** Survival curves of 9 of the 108 ‘hit’ isolates identified in the initial course screening of bacteria isolated in Crete against *Culex pipiens molestus* larvae. Data shows survival of mosquito larvae after exposure to live bacterial cell cultures over a period of 7 days relative to a control assay exposed only to Luria-Bertani medium.

**Supplementary Figure S1 Panel B:** Survival curves of 9 of the 108 ‘hit’ isolates identified in the initial course screening of bacteria isolated in Crete against *Culex pipiens molestus* larvae. Data shows survival of mosquito larvae after exposure to live bacterial cell cultures over a period of 7 days relative to a control assay exposed only to Luria-Bertani medium.

**Supplementary Figure S1 Panel C:** Survival curves of 9 of the 108 ‘hit’ isolates identified in the initial course screening of bacteria isolated in Crete against *Culex pipiens molestus* larvae. Data shows survival of mosquito larvae after exposure to live bacterial cell cultures over a period of 7 days relative to a control assay exposed only to Luria-Bertani medium.

**Supplementary Figure S1 Panel D:** Survival curves of 9 of the 108 ‘hit’ isolates identified in the initial course screening of bacteria isolated in Crete against *Culex pipiens molestus* larvae. Data shows survival of mosquito larvae after exposure to live bacterial cell cultures over a period of 7 days relative to a control assay exposed only to Luria-Bertani medium.

**Supplementary Figure S1 Panel E:** Survival curves of 9 of the 108 ‘hit’ isolates identified in the initial course screening of bacteria isolated in Crete against *Culex pipiens molestus* larvae. Data shows survival of mosquito larvae after exposure to live bacterial cell cultures over a period of 7 days relative to a control assay exposed only to Luria-Bertani medium.

**Supplementary Figure S1 Panel F:** Survival curves of 9 of the 108 ‘hit’ isolates identified in the initial course screening of bacteria isolated in Crete against *Culex pipiens molestus* larvae. Data shows survival of mosquito larvae after exposure to live bacterial cell cultures over a period of 7 days relative to a control assay exposed only to Luria-Bertani medium.

**Supplementary Figure S1 Panel G:** Survival curves of 9 of the 108 ‘hit’ isolates identified in the initial course screening of bacteria isolated in Crete against *Culex pipiens molestus* larvae. Data shows survival of mosquito larvae after exposure to live bacterial cell cultures over a period of 7 days relative to a control assay exposed only to Luria-Bertani medium.

**Supplementary Figure S1 Panel H:** Survival curves of 9 of the 108 ‘hit’ isolates identified in the initial course screening of bacteria isolated in Crete against *Culex pipiens molestus* larvae. Data shows survival of mosquito larvae after exposure to live bacterial cell cultures over a period of 7 days relative to a control assay exposed only to Luria-Bertani medium.

**Supplementary Figure S1 Panel I:** Survival curves of 9 of the 108 ‘hit’ isolates identified in the initial course screening of bacteria isolated in Crete against *Culex pipiens molestus* larvae. Data shows survival of mosquito larvae after exposure to live bacterial cell cultures over a period of 7 days relative to a control assay exposed only to Luria-Bertani medium.

**Supplementary Figure S1 Panel J:** Survival curves of 9 of the 108 ‘hit’ isolates identified in the initial course screening of bacteria isolated in Crete against *Culex pipiens molestus* larvae. Data shows survival of mosquito larvae after exposure to live bacterial cell cultures over a period of 7 days relative to a control assay exposed only to Luria-Bertani medium.

**Supplementary Figure S1 Panel K:** Survival curves of 9 of the 108 ‘hit’ isolates identified in the initial course screening of bacteria isolated in Crete against *Culex pipiens molestus* larvae. Data shows survival of mosquito larvae after exposure to live bacterial cell cultures over a period of 7 days relative to a control assay exposed only to Luria-Bertani medium.

**Supplementary Figure S1 Panel L:** Survival curves of 9 of the 108 ‘hit’ isolates identified in the initial course screening of bacteria isolated in Crete against *Culex pipiens molestus* larvae. Data shows survival of mosquito larvae after exposure to live bacterial cell cultures over a period of 7 days relative to a control assay exposed only to Luria-Bertani medium.

**2. Effects of culture time on insecticidal properties of five promising bacterial isolates**

In order to improve, as best possible, insecticidal metabolite output from bacterial isolates prior to extraction, a series of experiments were conducted to correlate length of time of standardised culture against insect mortality after exposure. For these assays, a selection of promising candidate bacteria were used, namely OTN1-C11, OTN72-C2, OTN78-C5, OTN82-C2 and OTN84-CD3.

Each of the bacteria were inoculated into 95 mL of LB broth and grown at 150 rpm and 27 ± 1 °C as in all other assays. Five replicate cultures were started at the same time, one of each of which was removed from the shaking incubator every 24 hours for a period of five days. After removal, cell counts were conducted using an Improved Neubauer Haemocytometer to determine if any relationship was present between cell concentration and the production of insecticidal metabolites. The remaining culture was then sonicated as according to the protocol in section 2.5 before bioassays were conducted again according to section 2.8. Mortality data at the 7 day experimental endpoint were analysed via 2-way ANOVA, using treatment and culture time as fixed factors, in SPSS v.22 (IMB Corporation, USA). Tukey’s post-hoc analyses were performed to determine the effect of culture time on mortality across the experiments.

**Supplementary Figure S2:** Cell concentrations of five representative bacteria grown over a range of 1-5 days. Data shows mean cell count per mL of culture medium, as calculated using an improved Neubauer haemocytometer.

Mortality data, shown below, demonstrated a low level of general correlation between cell concentration and insect mortality. Moreover, a range of differences were found between bacterial isolates peak production of insecticidal metabolites, demonstrating a long term necessity to fully elucidate upon the active metabolite and the optimal growth conditions for each individual bacterial isolate. Broadly, most of the cell cultures had stabilised after three days of culture time, and to avoid efficacy reduction from over-culture (as seen in OTN72-C2), a three day culture time was used for solvent extraction assays so as to broadly optimise insecticidal production for all isolates.

**Supplementary Figure S3:** Kaplan-meier survival curve of *Culex pipiens molestus* larvae exposed to sonicated cultures of *Chryseobacterium* OTN1-C11_Cr1 that were harvested after 1-5 days of total culture time. The figure shows the survival % larvae under each treatment throughout the course of the experiment. Controls consisted of an extracted LB dilution only. Errors shown are 95% CI.

OTN1-C11 was found to display the most rapid growth cycle of all bacterial isolates in these experiments; reaching above 9x10^5^ CFU mL^-1^ after just 48 hours, before stabilising between 8.58-9.28x10^5^ CFU mL^-1^ thereafter. Greater swings in log and death phases were noted in this isolate (Figure 1). Mortality was not considered to be dependent on culture length in this experiment, while differences were found between treatments and controls (F_(5, 45)_ = 441.379, p < 0.001), no differences in elicited mortality were found between any of the treatment groups (p = 1.000 in all cases), showcasing 100% mortality after a maximum of four days in all experiments.

**Supplementary Figure S4:** Kaplan-meier survival curve of *Culex pipiens molestus* larvae exposed to sonicated cultures of *Pseudomonas* OTN72-C2_Ps2 that were harvested after 1-5 days of total culture time. The figure shows the survival % larvae under each treatment throughout the course of the experiment. Controls consisted of an extracted LB dilution only. Errors shown are 95% CI.

OTN72-C2 was found to be grow more slowly than other bacterial isolates initially, producing around half of the concentration of cells per mL after 24 hours as compared to OTN1-C11. The end point concentrations were similar to most isolates stabilising at between 8.82 and 8.92 x10^5^ CFU mL^-1^ from 72 hours onwards (Figure 1). All culture times were found to produce strong insecticidal activity over controls (F_(5, 45)_ = 312.500, p < 0.001), and no significant differences in insecticidal activity were observed between bacteria grown for different periods (p = 1.000 in all cases) after 7 days. Interestingly, a longer period for mortality was observed when OTN72-C2 was grown for 5 days, potentially hinting at reduced efficacy over time. Further experimentation would be required to determine if insecticidal action were negatively correlated with bacterial culture time after 3 days.

**Supplementary Figure S5:** Kaplan-meier survival curve of *Culex pipiens molestus* larvae exposed to sonicated cultures of *Pseudomonas* OTN78-C5_Ps3 that were harvested after 1-5 days of total culture time. The figure shows the survival % larvae under each treatment throughout the course of the experiment. Controls consisted of an extracted LB dilution only. Errors shown are 95% CI.

OTN78-C5 was found to grow at a rapid exponential rate over the first 48 hours (Figure 1), before stabilising at 8.8-9.04 x10^5^ CFU mL^-1^ from 72 hours onwards. Significance was found between all groups (F_(5, 45)_ = 462.87, p < 0.001) and all culture times resulted in significant mortality over controls (p < 0.001 in all cases). Only 1-day cultures were found to produce lesser mortality than other treatments (p < 0.001 in all cases), and no significance differences in insecticidal efficacy were found between cultures grown for 2-5 days, due to 100% larval mortality when exposed to these cultures.

**Supplementary Figure S6:** Kaplan-meier survival curve of *Culex pipiens molestus* larvae exposed to sonicated cultures of *Bacillus* OTN82-C2_Ba1 that were harvested after 1-5 days of total culture time. The figure shows the survival % larvae under each treatment throughout the course of the experiment. Controls consisted of an extracted LB dilution only. Errors shown are 95% CI.

OTN82-C2 cultures were found to increase in cell concentration in a linear fashion over the 5 days (Figure 1); while the cultures reached similar concentrations per mL to OTN1-C11, OTN72-C2 and OTN78-C5 after 5 days, the initial growth phase was much more gradual, and peak concentration was reached only in day 5. This also correlated broadly with mortality, which was clearly affected by cell culture conditions (F_(5, 45)_ = 77.840, p < 0.001). As with other isolates, increases in mortality were correlated with daily increases in culture time (p < 0.001) up until 3-day cultures, which were not found to significantly differ to 4-day (p = 0.964) or 5-day (p = 0.910) cultures.

**Supplementary Figure S7:** Kaplan-meier survival curve of *Culex pipiens molestus* larvae exposed to sonicated cultures of *Stenotrophomonas* OTN84-CD3_St4 that were harvested after 1-5 days of total culture time. The figure shows the survival % larvae under each treatment throughout the course of the experiment. Controls consisted of an extracted LB dilution only. Errors shown are 95% CI.

OTN84-CD3 cultures were found to contain approximately 10 times less cells per mL after 5 days of culture as compared to the other isolates (Figure 1), and had broadly stabilised by day 3. With regards to mortality, the picture was somewhat different to other strains; overall significance was found (F_(5, 45)_ = 48.503, p < 0.001) between treatment groups. However, as opposed to most other strains, mortality was found to be more linear in terms of culture time. Significant increases (p < 0.01) in insect mortality were found with each additional day of bacterial culture prior to bioassay, with the exception of 4-day and 5-day cultures which were not found to elicit differing mortality to 3-day cultures (p = 0.872 and 0.504 respectively), largely due to the vast majority of the insects dying across both experiments. The above may suggest a gradual metabolite production that carries on accumulating after bacterial exponential growth phases.
